# Supplementary material for: Experiences and effect of implementing social health insurance (SHI) program in Nepal-A mixed method study
Source: PLOS Glob Public Health. 2025 Apr 24;5(4):e0003492. doi: 10.1371/journal.pgph.0003492 (PMC12021158; doi:10.1371/journal.pgph.0003492)
Supplement: S1 Table — (DOCX) [file pgph.0003492.s001.docx]

S1 Table. List of intervention and comparator districts

| **Province** | **Intervention Districts** | **Comparator districts** |
| --- | --- | --- |
| Koshi Province | Illam, Jhapa, Sunsari, | Dhankuta, Morang, Okhaldhunga, Panchthar, Sankhuwasabha, Taplegunj, Terhathum, Udayapur |
| Madhesh Province |  | Bara, Mahottari, Parsa, Rautahat, Saptari, Sarlahi, Siraha, Dhanusha |
| Bagmati Province | Bhaktapur, Chitwan, Makwanpur, Sindhuli, | Dhading, Dolakha, Kathmandu, Kavre, Lalitpur, Nuwakot, Rasuwa, Sindhupalchowk |
| Gandaki Province | Baglung, Gorkha, Kaski, Myagdi, Tanahu | Lamjung, Manang, Mustang, Nawalparasi, Parbat, Syangja |
| Lumbini Province | Palpa, Rolpa | Arghakhanchi, Banke, Bardiya, Dang, Rukum East, Gulmi, Kapilvastu, Parasi, Pyuthan, Rupandehi |
| Karnali Province | Jumla, Jajarkot, Surkhet | Dailekh, Dolpa, Doti, Humla, Kalikot, Mugu, Salyan, Rukum West |
| Sudurpaschim Province | Accham, Baitadi, Kailali | Bajhang, Bajura, Dadeldhura, Dharchula, Kanchanpur |
| ***Total: 73*** | ***Total Intervention: 20*** | ***Total Comparator: 53*** |
