# Supplementary material for: Experiences and effect of implementing social health insurance (SHI) program in Nepal-A mixed method study
Source: PLOS Glob Public Health. 2025 Apr 24;5(4):e0003492. doi: 10.1371/journal.pgph.0003492 (PMC12021158; doi:10.1371/journal.pgph.0003492)
Supplement: S1 Text — (DOCX) [file pgph.0003492.s002.docx]

**S1 Text. Data Collection Tools**

**Guide for Key Informant Interview (Overall)**

Questions:

1. Let’s begin by yourself. What is your current position? how long have you been working in this position? Have you been engaged in SHI program since the start or you joined later? Were you involved in any insurance program other than SHI?
2. In your opinion, what are the factors that contribute or hinder in SHI uptake? Why do you think people are enrolling/not-enrolling in SHI program?
3. Have you seen any difference in patient characteristics between SHI enrollees and non-enrollees? If so, what are the major differing characteristics?
4. In your experience, what are the challenges faced by health facilities to provide health services?

Probe for:

- Service location
- Availability of adequate infrastructure, resources- staffs, drugs, equipment
- Waiting time
- Providers motivation and satisfaction
- Trust between patient provider
- Timely reimbursements from insurance schemes
- Increased administrative workload

1. Have you found any improvement in these challenges after implementation of insurance program?
2. How is your experience so far with the insurance program? How has it affected or supported your current work?
3. Do you have any suggestions on how health facilities can enhance its services? What can be the role of Health Insurance Board in this?
4. Do you have anything else that you want to add?
5. Did you want to revisit any of the answers you gave me?

Thank you so much for meeting with me today.

**Guide for** **Key Informant Interview (specific for enrollment officer/assistant)**

Questions:

1. Let’s begin by yourself. What is your current position? how long have you been working in this position? Have you been engaged in SHI program since the start or you joined later?
2. In your opinion, what are the factors that contribute or hinder in SHI uptake? Why do you think people are enrolling/not-enrolling in SHI program?
3. Have you seen any difference in patient characteristics between SHI enrollees and non-enrollees? If so, what are the major differing characteristics?
4. How is your experience so far with the insurance program? What are the challenges you found in implementing the program?
5. Do you have any suggestions on how health insurance board can enhance its services?
6. Do you have anything else that you want to add?
7. Did you want to revisit any of the answers you gave me?

Thank you so much for meeting with me today.

**Guide for Focus Group Discussion with Insurance enrollees**

Questions:

1. What factors contributed to your decision on enrolling into health insurance program?

{Probe on:

- Where did you get the SHI related information: awareness program, FCHVs, friends/relatives, health workers, community leaders
- Past history of catastrophic illness (life-threatening or with threat of residual disability)
- Existing health condition (you or your family members)
- Foresee some health condition requiring treatment in future
- As a safety net

1. Tell us about your experiences while enrolling into insurance program.

{Probe for:

- Navigating the administrative system
- Getting and using the social security card
- Paying premium

1. Have you received any health care services after enrolling into the program? If so, how was your experience? Have you found any difference in services before and after enrolling into the program?
2. Have you considered continuing the insurance program?  If so, why?  What factors contributed to your decision to want to stay or to leave the program?

{Probe for: insurance scheme design features such as contribution rates, quality of health service provided

1. Do you have any suggestions on how the program can be improved?

That concludes our focus group.  Thank you so much for coming and sharing your thoughts and opinions with us.  If you haven’t already filled the sign-up sheet, please complete so. If you have any questions, please feel free to ask me or my friend here.

Sample Sign-up Sheet:

| Name | Age | Sex | Education | Insurance enrollment duration |
| --- | --- | --- | --- | --- |
|  |  |  |  |  |
|  |  |  |  |  |

**Guide for Focus Group Discussion- with Non-enrollees**

Questions:

1. Have you heard about SHI program? If so, where and from whom did you hear?

{Probe on:  awareness program, FCHVs, friends/relatives, health workers, community leaders or others

1. Have you ever thought about getting the insurance? Is so, why do you want to get the insurance?

{Probe on:

- friends/family members have insurance,
- Past history of catastrophic illness (life-threatening or with threat of residual disability)
- Existing health condition (you or your family members)
- Foresee some health condition requiring treatment in future
- As a safety net

1. What factors contributed to your decision on not getting insurance till now? Or why do think people are not getting insurance?

{Probe on:

- knowledge on SHI,
- navigating the administrative system of SHI
- family support
- lack of financial means to pay for premium
- don’t think it’s needed
- quality of hospital/health facility
- proximity of the health facility
- insurance scheme design features
- cultural norms
- trust to insurance program/health service providers included into SHI program

1. Do you have any suggestion for insurance program or government? How can they improve so that you will get insurance?

That concludes our focus group.  Thank you so much for coming and sharing your thoughts and opinions with us.  If you haven’t already filled the sign-up sheet, please complete so. If you have any questions, please feel free to ask me or my friend here.

Sample Sign-up Sheet:

| Name | Age | Sex | Education |
| --- | --- | --- | --- |
|  |  |  |  |
|  |  |  |  |

**Post** **Focus Group Survey Questionnaire**

As part of your participation in this focus group, we ask that you please fill out this brief questionnaire. Thank you!

1. What is your age?

……………………………years

1. What is your gender?

Male ...……………………………………………………☐1

Female ……………………………………………………☐2

Other ...……………………………………………………☐3

1. Which caste/ethnicity you belong to?

Brahman/Chhetri ………………………………………………….…☐1

Terai/Madhesi…. ………………………………………...☐2

Dalit ………………………………………. ☐3

Newar …………... ……………………………………………☐4

Janajati…………………….…………………………………………. ☐5

Muslim………… …………………………………...…. ☐6

Other………… …………………………………...…. ☐7

1. Are you head of household?

Yes ……………………………………………………☐1

No.……………………………………………………☐2   SKIP TO Question 6

1. What is the highest grade or year of school you attended?

Never attended school ………………………………………………….…☐1

Primary school (up to grade 5) …. ………………………………………...☐2

Secondary school (up to grade 10) ………………………………………. ☐3

High school or +2 …………... ……………………………………………☐4

Bachelor’s degree…………….…………………………………………. ☐5

Master’s degree or higher………… …………………………………...…. ☐6

1. What is your average annual HH income?

NPR………………………………………………………………

1. How many members are there in your family?

…………………………………………………………

1. Does any of your family member have chronic illness

Yes ……………………………………………………☐1

No…………………………………….………………☐2

1. Did any of your family member had catastrophic illness (life-threatening or needed care from specialized tertiary centers costing more than they could afford) in the past?

Yes ……………………………………………………☐1

No…………………………………….………………☐2

1. Do you have health insurance?

Yes ……………………………………………………☐1

No…………………………………….………………☐2 SKIP TO Question 14

1. Are all your family members insured?

Yes ……………………………………………………☐1

No…………………………………….………………☐2

1. If not, what is the age of the youngest member of your family who does not have health insurance?

……………………………………………………………years

1. How satisfied are you with the insurance program?

Very satisfied ……………………………………………………….…☐1

Somewhat satisfied ………………………………………………….…☐2

Not at all satisfied …………………………….…………………….…☐3

1. Where do you get your health care services/ under HI program? (Choose all that apply):

PHCC…………………………………………………………….….…☐1

Public hospital……………………………………………………….…☐2

Private hospital…………………………………………………………☐3

1. What services have you and/or your family members use at the hospital or PHCC? (Choose all that apply)

Outpatient care …………………………………………………………☐1

Inpatient care……………………………………………………………☐2

Emergency care…………………………………………………...….…☐3

Specialist care……………………………………………………….…☐4

Other………………………………………………………………....…☐5

None………………………………………………………………….…☐6

1. How satisfied are you with the health services you got from your primary health facility?

Very satisfied………………………………………………………….…☐1

Somewhat satisfied………………………………………………...….…☐2

Not at all satisfied…………………………………………………….…☐3

Thank you for taking the time to complete this questionnaire!

| FG ID #: | Moderator: |
| --- | --- |
| Date: | Venue: |
