# Supplementary figures and images for: Experiences and effect of implementing social health insurance (SHI) program in Nepal-A mixed method study
Source: PLOS Glob Public Health. 2025 Apr 24;5(4):e0003492. doi: 10.1371/journal.pgph.0003492 (PMC12021158; doi:10.1371/journal.pgph.0003492)

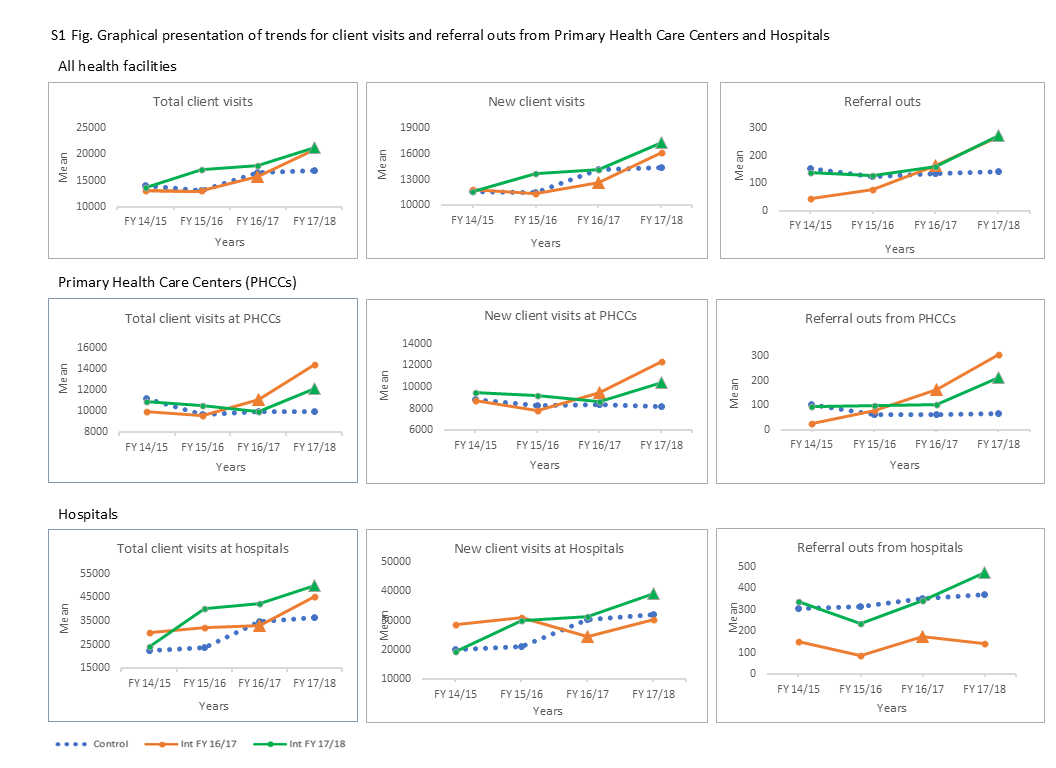

Supplement: S1 Fig — (TIF) [file pgph.0003492.s003.tif]
