# Supplementary material for: Experiences and effect of implementing social health insurance (SHI) program in Nepal-A mixed method study
Source: PLOS Glob Public Health. 2025 Apr 24;5(4):e0003492. doi: 10.1371/journal.pgph.0003492 (PMC12021158; doi:10.1371/journal.pgph.0003492)
Supplement: Attachment — Submitted filename: PGPH-D-24-00962_reviewer_3June.pdf [file pgph.0003492.s004.pdf]

PLOS Global Public Health

Experiences and effect of implementing Social Health Insurance (SHI) program in Nepal- A mixed method study.

--Manuscript Draft--

**Manuscript Number:**	PGPH-D-24-00962
**Article Type:**	Research Article
**Full Title:**	Experiences and effect of implementing Social Health Insurance (SHI) program in Nepal- A mixed method study.
**Short Title:**	Implementing SHI Program in Nepal
**Corresponding Author:**	Rajani Bharati<br>American Academy of Family Physicians<br>Leawood, KS UNITED STATES
**Order of Authors:**	Rajani Bharati
	Diana Romero
	Alexis Pozen
	James Sherry
	Bhuwan Paudel
	Mukesh Adhikari
	Prakash Acharya
**Keywords:**	Social Health Insurance; Nepal's National Health Insurance Program; Universal Coverage; Health Care Utilization; Mixed method study
**Abstract:**	Nepal initiated the National Health Insurance Program (NHIP) in 2016, but the enrollment rate is low, with an increasing dropout rate. This study examined the experience from the demand and supply side (service providers) with NHIP and its effect on insurance enrollment and health service utilization.<br>We employed an exploratory sequential mixed-method design, involving 14 focus group discussions and 20 key informant interviews, supplemented by quantitative data from Nepal's District Health Information System (DHIS-2).<br>We identified a complex interconnection between demand- and supply-side factors that influence the enrollment and renewal of the insurance program, as well as health-seeking behavior. The quality of health services played a pivotal role. Other contributing factors included inadequate awareness, limited geographical accessibility to health facilities, household income, perceived risk of getting an illness, and the perceived usefulness of health insurance programs. Conversely, the quality of services was contingent upon several challenges, such as increased patient flow and administrative burden without proportional resource growth, motivation of health service providers and enrollment assistants, difficulty in claims processing, reimbursement delays, cumbersome medicine procurement process, and insufficient information about the insurance program. Despite challenges, providers reported increased patient flow. Our data suggests an additional 9 and 32 daily client visits in Primary Health Care Centers and hospitals, respectively, corresponding to a 5% enrollment rate.<br>Our findings emphasize the need for a holistic approach, including better collaboration between the Health Insurance Board and the Ministry of Health and Population, policy reforms, community engagement, and improved service delivery to ensure equitable healthcare access.
**Additional Information:**	
**Question**	**Response**
**Financial Disclosure**	This paper is a result of a dissertation partially funded by the CUNY School of Public Health Dean's Dissertation Award 2019.

Powered by Editorial Manager® and ProduXion Manager® from Aries Systems Corporation

Enter a financial disclosure statement that describes the sources of funding for the work included in this submission and the role the funder(s) played. This includes grants and any commercial funding of the work or authors.<br><br>This statement will be typeset if the manuscript is accepted for publication.<br><br>*Please review the submission guidelines and the instructions link below for detailed requirements and guidance.*	
**Competing Interests**<br><br>On behalf of all authors, disclose any competing interests that could be perceived to bias this work.<br><br>This statement will be typeset if the manuscript is accepted for publication.<br><br>*Please review the instructions link below and PLOS Global Public Health's competing interests policy to determine what information must be disclosed at submission.*	The authors declare that they have no competing interests.
**Data Availability**<br><br>Before publication, Authors are required to make fully available and without restriction all data underlying their findings. Please see our PLOS Data Policy page for detailed information on this policy.<br><br>A **Data Availability Statement**, detailing where the data can be accessed, is required at first submission. Insert your Data Availability Statement in the box below.	DHIS 2 data are available at https://figshare.com/s/f94c28f2ad6988f28a17<br>Post focus group survey is available at https://figshare.com/s/2526de63ad89b2a1ec93<br>We are unable to provide qualitative interview/focus group transcripts.

Please see the data reporting section of our submission guidelines for instructions on what you need to include in your Data Availability Statement.<br><br>This statement will be typeset if the manuscript is accepted for publication.<br><br>*PLOS allows rare exemptions to address legal and ethical concerns. If you have legal or ethical restrictions, please use the box below to detail these in full sentences for the Journal team to consider.*	

Powered by Editorial Manager® and ProduXion Manager® from Aries Systems Corporation

Manuscript

Click here to access/download;Manuscript;SHI manuscript_5.2.24.docx

Full Title: Experiences and effect of implementing Social Health Insurance (SHI) program in Nepal- A mixed method study.

Short Title: Implementing SHI Program in Nepal

Author Name and Affiliation

Rajani Bharati * (Corresponding Author)
Current Address: American Academy of Family Physicians, Leawood, KS
At the time of study: CUNY Graduate School of Public Health and Health Policy
New York City, NY
Email: rbharati.ach@gmail.com
Phone: (929) 285 6858

Diana Romero
CUNY Graduate School of Public Health and Health Policy
New York City, NY
Email: diana.romero@sph.cuny.edu
Phone: (646) 364 9522

Alexis Pozen
Current Address: Unaffiliated
At the time of Study: CUNY Graduate School of Public Health and Health Policy
New York City, NY
Email: apozen@gmail.com

James Sherry
CUNY Graduate School of Public Health and Health Policy
New York City, NY
Email: james.sherry@sph.cuny.edu

Phone: (646) 364 0246
Bhuwan Paudel
Ministry of Health, Bagmati Province,
Kathmandu, Nepal
Email: bhuwan2000@hotmail.com

Mukesh Adhikari
Gillings School of Global Public Health
University of North Carolina, Chapel Hill,
North Carolina, US
Email: madhikari@unc.edu

Prakash Acharya
Missouri Heart Center
Email: prakashacharya.med@gmail.com
Phone: (282) 778 8859

Author Contributions

RB: Conceptualized and designed the project, conducted data collection and analysis, authored the manuscript, and served as the primary author.

DR: Acted as the dissertation committee chair, supported the IRB application, contributed to instrument design, supported data collection and analysis, and reviewed and edited the manuscript.

AP: Served as one of the dissertation committee members, assisted in instrument design, supported data collection and analysis, particularly in the quantitative section, and participated in manuscript review and editing.

JS: Also served as one of the dissertation committee members, provided support for project conceptualization and design, and participated in manuscript review and editing.

BP: Played a pivotal role in project execution in Nepal during their tenure with the Health Insurance Board. Helped adapt the data collection instrument for the Nepalese context, supported data collection, offered valuable insights into the insurance program, and contributed to manuscript review and editing.

MA: Assisted with data collection, particularly in the quantitative section, during their tenure at the Integrated Health Management Section of the Department of Health Services, Nepal. Also contributed to manuscript review and editing.

PA: Provided support throughout the project, including data collection, analysis, and manuscript review, and editing.

Abstract

Nepal initiated the National Health Insurance Program (NHIP) in 2016, but the enrollment rate is low, with an increasing dropout rate. This study examined the experience from the demand and supply side (service providers) with NHIP and its effect on insurance enrollment and health service utilization.

We employed an exploratory sequential mixed-method design, involving 14 focus group discussions and 20 key informant interviews, supplemented by quantitative data from Nepal's District Health Information System (DHIS-2).

We identified a complex interconnection between demand- and supply-side factors that influence the enrollment and renewal of the insurance program, as well as health-seeking behavior. The quality of health services played a pivotal role. Other contributing factors included inadequate awareness, limited geographical accessibility to health facilities, household income, perceived risk of getting an illness, and the perceived usefulness of health insurance programs. Conversely, the quality of services was contingent upon several challenges, such as increased patient flow and administrative burden without proportional resource growth, motivation of health service providers and enrollment assistants, difficulty in claims processing, reimbursement delays, cumbersome medicine procurement process, and insufficient information about the insurance program. Despite challenges, providers reported increased patient flow. Our data suggests an additional 9 and 32 daily client visits in Primary Health Care Centers and hospitals, respectively, corresponding to a 5% enrollment rate.

Our findings emphasize the need for a holistic approach, including better collaboration between the Health Insurance Board and the Ministry of Health and Population, policy reforms, community engagement, and improved service delivery to ensure equitable healthcare access.

Keywords: Social Health Insurance, Nepal’s National Health Insurance Program, Universal Coverage, Health Care Utilization, Mixed method study

Introduction

More than half of the world's population does not receive all the essential services they need [1]. In 2019, over a billion people worldwide spent at least 10 % of their household budget on health care and approximately 344 million people were pushed into extreme poverty because of unexpected out-of-pocket (OOP) health expenses [1].

Nepal has implemented various healthcare financing strategies over the past two decades, including user fees in public facilities, free healthcare policies, community-based insurance schemes, and several non-governmental initiatives at the community level [2]. However, these interventions were fragmented, provided insufficient financial protection, and often did not meet the target groups' medical needs [2].

Between 2010 and 2017, the Government of Nepal spent 6-8% of its GDP on healthcare, yet the OOP spending increased from 43% to 60% [3–5]. To address this issue, the Government of Nepal introduced the National Health Insurance Program (NHIP) in 2013, aiming to achieve universal health coverage by 2030 [2]. The primary goals of this program were to ensure equitable access to quality health services, financial protection against catastrophic illness, strengthen the health system, and improve the health-seeking behavior of the public [2].

Initially launched in three pilot districts in 2016 (Kailali, Baglung, and Illam), the NHIP went through a staggered expansion and has reached 75 districts [6]. The key features include an autonomous governing body, single risk pool, gatekeeping system in the service delivery, family-based membership, DRG payment system to providers, and subsidization of premiums to certain vulnerable groups [7] (See Table 1).

Table 1. Nepal's National Health Insurance Program Design Features

NHIP design features [7]	***Details***
**Governing body**	Health Insurance Board, an autonomous body
**Legal Framework**	Health Insurance Act 2017
**Population coverage**	Voluntary enrollment at present.<br>However, the Health Insurance Act 2074 has envisioned universal enrollment
**Financing and membership**	Family-based membership and premium contribution<br>Employee contribution (yet to be started)<br>Complementary funding from the government's budget
**Contributions**	NPR 3500 (USD 3.5) per annum for HH of 5 members<br>NPR 700 per additional member<br>Salary contribution: 1% of base salary for each employee and employer (yet to be started)<br>Subsidy for certain categories
**Subsidies on contribution amounts**	100% subsidy- families with ultra-poor identification cards<br>100% subsidy- families with members who have complete disability, leprosy, HIV, and multidrug-resistant TB;<br>100% subsidy- senior citizen (70 years and above)<br>50% subsidy- Female Community Health Volunteers (FCHVs)
**Registration and Service start date**	Four fixed service start dates in a year. There is a lag time of one to four months between registration and service start date. For instance, those registered or renewed between January 16 and April 15 will have a service start date of May 15.
**Risk pooling and risk protection**	Single fund with no fragmentation

**Benefit package**	Health promotion, prevention, treatment, rehabilitation, and ambulance service package with a benefit ceiling of NPR 100,000 for a family of five per year.<br>An additional benefit of NPR 20,000 for each added member not exceeding a maximum of NPR 200,000 per family.<br>Senior citizens get a separate benefit amount of up to NPR 100,000.<br>Ambulance service only covers emergency conditions
**Health care delivery**	Both public and private health facilities with gatekeeping<br>Only public facilities are allowed to serve as a "Primary service point". Enrollees choose their primary service point during registration.<br>Any facilities that are not primary service points can provide only emergency and referral services.<br>Referral cards are only valid for seven days.<br>HIB enters into contractual agreements with health facilities, which is valid for five years.
**Payment to providers**	Capitation, per diem, and case-based (Diagnosis Related Groups)<br>HIB pays providers based on various packages and individual items.<br>The packages include Diagnosis based, per day basis, OPD, Emergency, Eye hospital outpatient/emergency, cardiac services, Surgical, and others.<br>The same payment rate for public and private facilities<br>HIB does not pay if health facilities are providing services under other vertical programs such as free health programs, safe motherhood programs, and family planning programs.
**Remuneration to Enrollment Assistants (EAs)**	10 to 15% of the premium amount depending on the performance.

**Claim management and Information System**	Integrated Management Information System (IMIS)

Enrollment into NHIP has been a significant challenge, especially as it started with voluntary enrollment among informal sectors, a practice that continues today [8]. Initially, the enrollment rate averaged around five percent in the program's first two years [9–12]. However, subsequent policy changes, such as introducing subsidies for senior citizens and other vulnerable groups led to an increased enrollment rate of approximately 17% in 2019/20 [9–12]. Notably, Ranabhat and colleagues reported that at least one-third of the total enrollment consisted of subsidy enrollments[13]. Dropout rates also emerged as a significant concern, with national rates of 44.5% and 38.4% in 2017/18 and 2018/19, respectively [11,12]. In Kailali district alone, dropout rates reached 77% in FY 2017/18 [11].

With an increased enrollment among high-risk groups and a high dropout rate among presumably healthy populations, understanding the determinants of insurance enrollment becomes crucial. Additionally, some tertiary-level public hospitals have not participated, foreboding service providers' issues. Few studies have explored the NHIP's demand-side determinants [14,15] or has assessed its impact on healthcare utilization [16]. This study aims to gain a holistic insight from both the demand and supply side (service providers), exploring perceptions of enrollees and non-enrollees, challenges faced by service providers, and NHIP's impact on health service utilization. Specific aims include:

1. Explore the demand side (enrollees and non-enrollees) determinants affecting insurance enrollment.

2. Understand the service providers' experiences and challenges in providing health services under the NHIP.
3. Assess NHIP’s effect on health service utilization, including ‘total client visits,’ ‘new clients visits’, and ‘referral outs.’

Materials and Methods

Study design: The study utilized an exploratory sequential mixed-method design [17,18]. Qualitative data were collected initially, revealing an increase in patient visits and referrals at health facilities. Subsequently, quantitative data was collected to further examine healthcare utilization, specifically total client visits, new client visits, and referral outs, aligning with the qualitative findings. Focus group discussions and Key Informant (KI) interviews were used to explore the perceptions and experiences of insurance enrollees, non-enrollees, and service providers. The difference-in-difference (DID) approach was used to assess the NHIP’s impact on health service utilization.

Study setting, population, sampling, and recruitment: The qualitative data was collected from two NHIP pilot districts- Baglung and Kailali, ensuring representation of urban and rural settings. KIs included Health Insurance (HI) focal persons, physicians, nurses, medical administrators, Enrollment Officers (EOs), and Enrollment Assistants (EAs) from PHCCs, district/regional government hospitals, private hospitals, and province/district health insurance offices. The focus group participants included health insurance enrollees, ex-enrollees, and non-enrollees. Ex-enrollees, who had experience with the program but did not renew their membership, were grouped with enrollees for discussions. Anyone who could engage in constructive discussions about NHIP was purposively selected for the study.

DHIS-2 data [19] from all the health facilities in 73 out of 77 districts of Nepal were used for the quantitative section of this study. Four districts were excluded as they started insurance service late in FY 2017/18. The 20 districts that started the insurance program in 2016 and 2017 were regarded as intervention districts; 53 districts that had not started the program or started after May 2018 were categorized as non-intervention districts. (See S1 Table). Since only public health facilities are mandated to report on DHIS-2, the data do not include medical colleges, private hospitals, and tertiary centers. In the final analysis, we used 240 health facilities in FY 2014/15 (pre-intervention) and 251 facilities in FY 2017/18 (post-intervention). (See Fig 1).

Fig 1. Flowchart showing the selection of districts and health facilities.

Data collection and analysis: The data was collected between 25/11/2019 to 10/01/2020. 23 key informant interviews (87% response rate), 14 focus group discussions (six in Baglung and eight in Kailali district), and post-focus group surveys with focus group participants were administered using the semi-structured interview guides and questionnaires. (see S2 File) All the instruments were translated into the Nepali language. In-person interviews were mainly held in the office setting of health facilities, province/district HI offices, or at EAs house. Focus groups were conducted at health facilities, EA's or community social workers' houses, and school/temple yards.

Each interview and focus group were audiotaped, transcribed, and translated into English. The data were analyzed using a grounded theory approach [20]. Two coders thoroughly reviewed the transcripts. The first coder reviewed all the transcripts and set the initial codes, while the second coder reviewed 25% of the transcripts (representative of all subgroups) and assigned the developed codes to the transcripts. The initial inter-coder reliability for interview data was 97%, and the focus group was 96%, which was increased to 100% by sorting, synthesizing, and integrating the codes into sub-themes and themes. The coding process continued until new interview/focus group data

did not alter the definition or score of the codes. Dedoose software was used for the coding, memoing, and analytical functionality [21]. After the focus group session, post-focus group survey was conducted with participants. The survey data was then analyzed using Microsoft Excel to compare the demographic characteristics of enrollees and non-enrollees.

The quantitative data was obtained from the DHIS-2 (Nepal's national health information management system) through the Integrated Health Information Management Section under the Management Division, Department of Health Service (DHS) Nepal. Regarding the analysis, trends of the mean values of outcome variables were examined for four consecutive years, starting from FY 2014/15 to FY 2017/18, to visually inspect the parallel trend. Once the assumption of the parallel trend for Difference in Difference (DID) was met [22,23] (See S3 Figure), the DID estimation was made to calculate the average treatment effect of the NHIP comparing implementation and comparator districts between FY 2014/15 and FY 2017/18.

We used the DID model: [24]

$$Y_{hgt} = \alpha + \beta Post_t + \lambda Treatment_g + \delta\left(Post_t * Treatment_g\right) + \varepsilon$$

Here, g indexes the group (g=0 for the comparison district, 1 for the implementation district), and t represents time (0=2014/15, 1=2017/18).

Y is the outcome of interest for time t and health facility h in group g. Alpha (α) is the intercept. Beta (β) represents the parameter for the time component (pre- or post-intervention). Lambda (λ) represents the parameter for the group fixed effects. Delta (δ) *represents the interaction between time and group effect, which is the parameter of interest and is called the difference-in-difference estimator. It estimates the average treatment effect of the policy (the effect of treatment on the average*

outcome of Y) for districts that implemented the health insurance program compared to those that did not. Epsilon (ε) is the error term.

The outcome variables included total client visits, new client visits, and referral outs. We also conducted a subgroup analysis based on the types of health facilities (hospitals and PHCCs). The HIB aimed to balance the number of implemented districts among the provinces while conducting staggered implementation. Therefore, we did not include any time-varying district-level control, assuming that the districts included in the intervention groups closely resemble the districts in the control group. Additionally, we did not incorporate facility-level covariates into the model, such as the availability of resources, due to a large number of missing data in DHIS-2.

We removed the 19 facilities with missing values for outcome variables from the analysis (15 in pre-intervention and four in post-intervention years). The facilities with missing values were spread across the country and in both intervention and non-intervention districts. None of the districts had more than one facility with missing data.

All the statistical analyses for quantitative data were conducted using STATA 14.0 software [25].

Ethics statement

Ethical approval was obtained from the Institutional Review Board (IRB) of the City University of New York (CUNY) (protocol # 2019-0537) and the Nepal Health Research Council (review # 568/2019). We explained the study objectives, benefits, risks, right to refuse participation, and confidentiality of responses to all the participants. Both verbal and written informed consent were obtained from the participants.

Results

A total of 97 individuals participated in 14 focus group discussions, of which 49 were insurance enrollees and the remaining 48 were non-enrollees. Nearly all participants also completed the post-focus group survey questions. Most focus group participants were female and had an education below high school. The mean age of participants was 45 years for enrollees and 37 years for non-enrollees. The proportion of respondents who were Dalits (a historically marginalized ethnic group in Nepal), had higher annual income, and used private health facilities explicitly were higher among the non-enrollee group. In contrast, the proportion of those with chronic illness in the family or a history of catastrophic illness in the family was higher among the enrollee group. (See Table 2)

Table 2: Demographic and health-related characteristics of focus group participants

Characteristics	***Insurance enrollees***	***Insurance non-enrollees***
Districts	*N= 49*	*N=48*
Baglung	23 (47%)	19 (40%)
Kailali	26 (53%)	29 (60%)
Age (in years)	*N= 49*	*N= 48*
(mean/SD)	45 yrs (12)	37 yrs (13)
Sex (N/%)	*N= 49*	*N= 48*
Male	12 (25%)	3 (6%)
Female	37 (75%)	45 (94%)
Caste/ethnicity (N/%)	*N= 49*	*N= 48*
Brahman/Chhetri	29 (59%)	22 (46%)
Janajati	12 (25%)	10 (21%)
Dalit	8 (16%)	16 (33%)
Head of Household (N/ %)	27 (55%)	16 (33%)
Education	*N= 49*	*N=48*
Never attended school	20 (41%)	14 (29%)
Primary School (up to grade 5)	12 (24.5%)	14 (29%)
Secondary School (up to grade 10)	12 (24.5%)	10 (21%)

High School or +2	4 (8%)	9 (19%)
Bachelor's degree	0	1 (2%)
Master's degree or higher	1 (2%)	0
Annual income (in NRP)	*N= 32*	*N= 36*
(mean/SD)	257,383 (173,339)	221,666 (397,179)
Family size (number of members)	*N= 49*	*N=48*
(mean/SD)	6 (3)	5 (2)
Chronic illness in family members (N/%)	30 (61%)	20 (42%)
History of catastrophic illness in the family (N/%)	27 (55%)	19 (40%)
Enrollment of all family members in insurance (among enrollees)	35 (73%)	NA
The average age of the youngest family member that is not enrolled in the insurance program (among enrollees)	11 years (8)	NA
Satisfaction with the insurance program	N=48	NA
Very Satisfied	7 (15%)	
Somewhat satisfied	33 (69%)	
Not satisfied at all	8 (16%)	
Facilities where they primarily obtain health services	N=49	N=48
Government facilities only	37 (76%)	27 (56%)
Private facilities only	2 (4%)	11 (23%)
Both government and private facilities	10 (20%)	10 (21%)
Type of services used under the insurance program	N= 46	NA
Outpatient care only	23 (50%)	
Other services such as inpatient, emergency, and specialist care, including outpatient care	23 (50%)	
Satisfaction with primary health centers under the Insurance program	N=46	NA
Very satisfied	5 (11%)	
Somewhat satisfied	30 (65%)	
Not satisfied at all	11 (24%)	

Among the 23 key informants, most (n=17) were males. The largest percentage of informants were affiliated with hospitals (55%), followed by PHCCs (25%) and HI province/district offices (20%).

Key Informants and focus group participants described various factors affecting enrollment and renewal in the NHIP. Rather than being discrete, these factors were found to be overlapping and intricately connected. The interaction between these factors was varying, with some factors being mutually dependent while the others being a consequence of each other. We have tried to capture this complex relationship using a Fishbone diagram (See Figure 2). The details are described below.

Figure 2. Fishbone diagram showing the relationship between various demand and supply side factors in Health Insurance enrollment and health service utilization.

Quality of health services played a central role. The bottom line for the majority was that if they receive good health care, they will enroll into the program. People considered the availability of resources (medicine, skilled workforce, diagnostic services), well-behaved (i.e., respectful) service providers, short wait times, and clean health facilities that are easy to navigate as necessary elements for quality health services.

Availability of medicine and diagnostic facilities: The unavailability of medicine was the primary cause of discontentment among insurance enrollees, leading to non-renewal. Key informants outlined various reasons for the unavailability of medicine, such as tedious procurement system, delays in reimbursement from HIB, difficulties in timely convening Health Facility Operation and Management Committee (HFOMC) meetings, frequent absence of medical officers required for purchase approval, and shortage of essential medicines under free health services (which are not covered by HIB).

Many enrollees reported disparities in medicine provision as one stated, "*They provide different medicine for non-enrollees and cheap, low-quality medicine for us. Even if medicine is available,*

they withhold it once we show our insurance card." (Enrollee, FG 13). Several providers acknowledged maintaining different medicine sets and often offering the cheapest options to enrollees due to bidding processes favoring low-cost contracts under NHIP. Additionally, HIB encourages using generic medicine and has a fixed payment rate. Facilities may prioritize cheaper medicine procurement to maximize profits. Mistrust with NHIP medicines further escalated due to discrepancies between prescribed brand names and dispensed medicines. Some physicians were unaware of the need to prescribe generic names, while others hesitated to use generics, citing perceived differences in efficacy depending on the brand.

The lack of diagnostic facilities significantly influenced referrals, as people were reluctant to spend time navigating multiple health facilities for appropriate treatment. While some facilities reported acquiring diagnostic equipment after enrolling in the NHIP program, there were complaints about insufficient staffing and a lack of skilled personnel to utilize these resources effectively as evident in the following statement. "*Only I can operate the X-ray device, but with increased patient load and documentation requirement, I hardly have any time to operate the machine*" (PHCC In charge 2).

Availability of service providers and their behavior: Many focus group participants mentioned the unavailability of skilled health workers and their unpleasant behavior as their prime reason for avoiding public health facilities. One participant expressed frustration, stating, "*We rarely see doctors, and we are not even talking about specialties... we never receive prior notice about their leave. We wait in line for hours and then find out the doctor is on leave*" (Non-enrollee, FG 4).

Service providers in public [illegible]lities expressed concerns about insufficient staffing and infrastructure to match the increased patient flow and administrative workload. PHCCs, for instance, typically have only one sanctioned doctor's position, which often remains unfilled. Some

PHCCs with robust HFOMCs have lobbied Village Councils to hire new staff and provide training to existing staff. However, the high staff turnover has resulted in frequent training needs, often resulting in the prolonged absence of staff from health facilities. Furthermore, there is a lack of sanctioned positions, such as pharmacist or administrative assistants, necessary to meet NHIP requirements.

Some front-line service providers expressed their frustration in dealing with beneficiaries' misconceptions and expectations. A few reported facing numerous "unreasonable" demands, including requests for fruit juices, unnecessary referrals to higher centers, and transportation costs from insurance enrollees. One provider voiced their concern, saying, "*We are already overwhelmed by increased patient flow and do not have time to address the misunderstandings. When people start complaining and arguing, it becomes very difficult to maintain a calm demeanor*" (Physician, 1).

Wait time: Participants reported long wait times as a significant deterrent to choosing public health facilities. Wait times were already an issue in public health facilities, and they worsened with an increased patient flow (approximately between 20-70% post-NHIP adoption) and constricted time for patient care caused by administrative burdens.

The NHIP's requirement for establishing a primary contact point and need for referral has amplified the patient flow in public health facilities. Furthermore, the referral card remains valid for only 7 days, necessitating patients to obtain a new referral for follow-up visits after being discharged from referral centers. Patients must be physically present to receive a referral, as service providers claim they cannot process claims without examining patients. One participant shared, *"It took me over seven days to arrange and secure an appointment at the tertiary health center and my referral card expired by then. Since I had to return to village to obtain another referral*

card, which I couldn't manage, I couldn't use my insurance card" (Enrollee, FG 3). Although the patient flow has significantly increased in public facilities, particularly PHCCs, the number of individuals actually receiving services remains relatively low. As one informant explained, "*Lately we don't have doctors. So, we mostly have referral cases. We refer 15-20 patients in a day"* (PHCC In charge- 3). Additionally, patients often request referrals specifically for private facilities, as private facilities can only take referrals or emergency cases.

Many providers reported increased administrative burden due to the new IMIS reporting requirement and lack of connectivity with other systems like DHIS-2. Some found the claim system confusing, especially for claiming multiple diagnoses and unforeseen complications.

Private facilities did not face issues with resource availability or wait times but they reported charging insurance enrollees the difference (gap amount) between NHIP coverage and their rate. A representative from a private hospital stated, "*The rate provided by HIB is too low. We either offer inadequate care or face financial difficulties. Providing subpar care would harm our reputation, so we charge patients to cover the cost difference.*" (HI focal person, private facility, 1). Unfortunately, this situation has led to misunderstandings about NHIP's promise of no copayments.

Choice of health facilities depended primarily on the perceived health service quality, geographic accessibility, and facilities' ability to cater to special populations like children, the elderly, and individuals with disabilities. Many people opt for private facilities or seek treatment in neighboring Indian facilities, especially in border districts. This preference reduces the demand for NHIP since only select private facilities accept insurance, and coverage is limited to emergencies and referrals. Several other factors also influence this choice including proximity to primary service points, the

higher opportunity cost of seeking treatment at government facilities with long wait times, and service provider behavior.

Lack of awareness about NHIP was another significant factor influencing enrollment. Despite efforts through mass media, many non-enrollees were unaware of the program's existence. Some EAs with social standing were motivated to conduct door-to-door visits. However, others primarily engaged with individuals who contacted them directly for enrollment, avoiding efforts to reach the uneducated, lower-income, and hard-to-reach population due to the time-consuming nature of persuasion and low compensation. One EA mentioned, *"We have to spend atleast about 5 days to get one family enrolled and get paid only NPR 300 for five days' worth of work."* (EA 1).

Moreover, there was a lack of trust in EAs, with many perceiving them as mere "*insurance agents*." Healthcare providers, usually trusted sources, lacked sufficient knowledge and time to provide information. The absence of insurance registration services at health facilities further compounded the issue.

For existing insurance enrollees, health insurance literacy played a significant role in the renewal process. The lack of comprehensive information resulted in misunderstandings, leading to discontentment with the program. One participant expressed disappointment, stating, "*When enrolling us, they assured that everything would be covered. But the health facility denied reimbursements. So, I did not renew the insurance anymore."* (Enrollee, FG 9). This discontentment not only impacted renewal rates but also triggered negative word-of-mouth, dissuading potential new enrollees as one participant remarked "*No one has shared a positive experience. Even the insured people do not recommend that we enroll*" (Non-enrollee- FG 4).

Ability to pay for premiums Despite subsidies for the poor, low-income individuals had low enrollment due to ineffective means-testing. Participants reported that the poverty list did not accurately identify the real poor people and emphasized that EAs had not reached out to poor people. Conversely, EOs and EAs expressed apprehension about using the poverty list, as one explained, "*I witnessed a community dispute arising from the poverty list and I don't want to entangle with that.*" (Enrollment Officer-1).

The **perceived risk of getting ill** played a significant role in enrollment. Primarily, individuals with chronic illnesses and those requiring regular medications were inclined to enroll in the program. However, enrollment was often dependent on the availability of medicine at the primary service center. Families with more than five members typically refrained from enrolling those who were less likely to get sick, usually the youngest members. Interestingly, the possibility of catastrophic illness or accidents did not motivate people to enroll in the program, likely due to the reported poor service quality of public health facilities as one participant elucidated, "*Although I am insured, I might not use it during acute events like accidents or emergencies due to the significant delays in receiving service at government facilities*" (Enrollees, FG 12).

Perceived usefulness of health insurance varied among KIs, insurance enrollees, and non-enrollees. While some KIs appreciated the concept, many doubted its effectiveness in reaching vulnerable populations and the health systems' readiness. A respondent shared, *"The health institutions have not taken it as an opportunity but as a liability. If the health system functions properly, insurance is a nice program. However, our health facilities are not up to mark"* (HI focal person, 8).

Many enrollees and non-enrollees often did not perceive health insurance as useful considering it unnecessary for minor illnesses, especially with the availability of concurrent free health service

programs and alternative financing mechanisms (public service employees and uniformed service employees, private-sector employees). Some participants see it as a waste of money if they do not spend enough on treatment to match their premium. The absence of major tertiary referral centers and private hospitals as service providers further diminished the program's usefulness. Concerns also arose about the lag time in service provision, particularly regarding reactivation if enrollment expired, with waiting periods lasting 1 to 4 months.

As many focus group participants complained of overcrowding, and Key Informants reported a substantial increase in patient visits after adopting NHIP, we examined the treatment effect of policy on patient visits between intervention and comparator districts using the DID method. The findings (see Table 3) suggested an increase in the average number of total and new client visits by 4,729 and 2,721, respectively, in overall health facilities of intervention districts compared to comparator districts, due to the policy's effect. Similarly, the average number of referral outs increased by 163 in intervention districts. However, referral outs were only statistically significant for PHCCs. In subgroup analysis, we found a statistically significant increase in client visits and referrals for PHCCs but not for hospitals. Nonetheless, the findings indicated an increase in client visits by an average of 9 clients per day in PHCCs and 32 clients per day in hospitals.

Table 3: Difference in Difference estimation

**Variable**	**Interaction co-eff**	**95% CI**		**p-value**
**All health facilities**				
Total client visits	4729	-3801	13259	0.28
New client visits	2721	-4207	9649	0.44
Total referral outs	163	58	268	0.01*
**Sub-group analysis**				
Hospitals				
Total client visits	10072	-20527	40671	0.52
New client visits	5065	-20363	30493	0.69
Total referral outs	39	-212	290	0.76

PHCCs				
Total client visits	3051	-731	6833	0.1**
New client visits	2031	-306	4368	0.09**
Total referral outs	192	88	296	<0.001*

*H_0: the means are not different. *P<0.05: reject null hypothesis at 5%. **P<0.1: reject null hypothesis at 10%.*

Discussion

This study found a complex relationship between demand and supply factors affecting insurance program enrollment and healthcare utilization. The perceived quality of health services and proximity to facilities played a central role, often influenced by various NHIP design features and implementation issues like claim difficulties, delayed reimbursements, cumbersome medicine procurement, and the requirement of primary service points, among others. Factors such as lack of awareness, perceived risk of illness, and insurance program usefulness also played roles.

NHI[illegible] design closely resembles the Social Health Insurance Systems model by Providing for Health (P4H), a partnership between France, Germany, ILO, WHO, and the World Bank.[26] However, it is crucial to note that the context and implementation processes vary among countries, subsequently leading to different outcomes in universal health coverage. Our findings suggest the following implications.

Adverse selection: Despite policy changes, voluntary enrollment continues in NHIP. A study found that approximately one-third of the current enrollees receive subsidies,[13] consistent with our qualitative assessment indicating higher enrollment of older individuals and dropout of healthier ones. While Family-based membership helps,[27] our post-focus group assessment revealed that a quarter of households only partially participate, often enrolling high-risk or chronically ill

members. Though our focus group sampling may lack causal inferences, comparable instances of partial enrollment exist in other countries,[28] and several studies indicate chronic illness as a predictor for insurance enrollment in Nepal.[14,15]

Enforcing mandatory enrollment could address adverse effects, but it is challenging. The Philippines achieved high population coverage despite voluntary enrollment, but nearly half of those enrollees were subsidized, raising sustainability concerns.[27,29] Nepal is pursuing a similar strategy with free and subsidized premiums for certain groups, albeit without revenue streams like the Philippines' "sin tax".[29] Issues such as false wage reporting, evasion of registration, and employer non-compliance can arise even in the formal sector enrollment.[30] Korea achieved universal coverage with mandatory enrollment, mostly due to its higher economic growth.[30] However, Nepal's fluctuating GDP growth[31] makes this approach challenging. Alternatively, Thailand's tax-based system presents an option but requires steadfast political commitment and a robust healthcare delivery system, particularly in disadvantaged areas,[32] which is also a challenge for Nepal.

*Financial risk protection***:** One of the key objectives of social health insurance entails providing financial risk protection against catastrophic health expenditures, which necessitates balancing a comprehensive benefits package, coverage of the poor, and financial sustainability.[33,34] A preliminary estimate from Columbia suggests that achieving this may cost close to 20 percent of the GDP,[34] a figure beyond Nepal's economic capacity. Currently, NHIP offers modest benefits, with a benefit ceiling of NPR 100,000 for a family of five members, and faces challenges such as non-acceptance by many non-profit service providers.

Despite its gate-keeper function, our qualitative findings suggest that many individuals primarily use primary-care facilities for referrals, often opting for private hospitals. While referral to private

facilities might help with access to care, one needs to note that private facilities are charging the gap rate to the insurance enrollees and NHIP lacks a clear statement prohibiting this practice. This could inflate the OOP spending, mirroring the situation in the Philippines where insured and uninsured patients faced comparable costs due to increased price-cost margins to insured patients.[35] Moreover, many insurance enrollees were reluctant to use public facilities for severe illness due to wait times and service quality concerns, diminishing the perceived value of the insurance program.

Fund shifting from public to private entity: Gertler and Strum estimated a 33 percent reduction in public delivered care in Jamaica after expanding SHI.[36] We do not have such an estimate for Nepal, but the annual report from HIB indicates a large portion of reimbursement goes to the private facilities.[12] It signifies the shifting of public funds to the private entity even though SHI's objective is to reduce financial pressure on public budgets.[37]

Inequitable access to health care: Nepal is trying to provide equitable access to care by subsidizing premiums to poor and other disadvantaged groups. However, it has not been effective due to insufficient means-testing and health facilities' inability to cater to needs. Besides, the insurance program is expanded only up to the PHCC level, and most of the referral health facilities are centered around the cities. This raises a concern about equity for the rural population and portends that the insurance program might transfer funds from rural disadvantaged people to more affluent urban populations.

Increase in health care utilization: Both qualitative and quantitative findings suggest the increase in overall patient flow in health facilities with the health insurance program. Our quantitative analysis reaffirmed claims of increased patient flow in health facilities, revealing an average increase of 9 patient visits per day in PHCCs and 32 patient visits per day in hospitals. This aligns

with a separate study indicating a utilization rate of 88.7%.[16] Notably, this increase was observed when the enrollment rate to the insurance program was only around 5%.[9–12] The lack of concurrent increase in sanctioned posts for health service providers indicates the need for additional resources; without which the quality of care may further deteriorate as enrollment rate grows.

Strengths of the study: The study's design provided rich insights into participants' experiences. While our sampling is purposive, we incorporated perspectives from diverse participants across various geographic regions, reaching data saturation. Furthermore, our findings are consistent with other studies both within and outside Nepal. We also provided a preliminary assessment of health service utilization using national health system data (DHIS-2), which is a novel approach.

Limitations of the study: While this study explored the experiences of beneficiaries and providers, the perspective of the purchaser (i.e., Health Insurance Board), was not included. Many tertiary-level health facilities either do not participate in or withdraw from the insurance program, requiring in-depth exploration. Our quantitative analysis findings should be treated as preliminary. Due to missing data in DHIS-2, we couldn't account for facility-level confounders such as the availability of resources. Notably, these covariates may serve as mediators rather than confounders, given that NHIP's objective is to enhance health care quality. Data from private and tertiary referral centers were lacking, as they are not mandated to report to DHIS-2, potentially underestimating the NHIP's overall impact. Our study also lacks an estimate of adverse selection, financial protection, or equitable access to care, necessitating further investigation.

Conclusion

This study highlights the complex relationship between demand and supply factors affecting health insurance enrollment and subsequent health service utilization. Perceptions of service quality, including resource availability, play a crucial role in enrollment and renewal decisions. Service provision challenges and provider behavior contribute to beneficiary dissatisfaction, necessitating improved service delivery and provider training. Accessibility issues, long waits, and referral complexities drive individuals to private healthcare and out-of-pocket payments, undermining the insurance program's value. Lack of awareness and financial concerns hinder enrollment efforts, emphasizing the need for targeted outreach and community engagement. Despite these challenges, healthcare utilization has increased significantly post-implementation. Ensuring quality healthcare under the insurance program is vital for retaining enrollees and motivating non-enrollees to join. Addressing these challenges requires a holistic approach, including improved coordination between MOHP and HIB, policy reforms, community engagement, and enhanced service delivery to ensure equitable access to quality healthcare.

Acknowledgements

We extend our gratitude to the CUNY Graduate School of Public Health and Health Policy, the Nepal Health Insurance Board, including its province and district offices in Kaski, Baglung, and Kailali, as well as the Integrated Health Management Section of the Department of Health Services Nepal. Special thanks go to the Enrollment Assistants, health service providers, and the community people from Kailali and Baglung Districts of Nepal.

References

1. Tracking universal health coverage: 2023 global monitoring report [Internet]. Geneva: World Health Organization and the International Bank for Reconstruction and Development / The World Bank; 2023 [cited 2024 Mar 15]. Available from: https://www.who.int/publications/i/item/9789240080379

2. Ministry of Health and Population Nepal. National Health Insurance Policy 2013. Ministry of Health and Population Nepal; 2013.

3. Witter S, Khadka S, Nath H, Tiwari S. The national free delivery policy in Nepal: early evidence of its effects on health facilities. Health Policy Plan. 2011 Nov 1;26(suppl_2):ii84–91.

4. World Health Organization. Health Financing Profile 2017 [Internet]. World Health Organization; 2017 [cited 2018 Nov 20]. Available from: http://apps.who.int/iris/bitstream/handle/10665/259643/HFP-NEP.pdf?sequence=1&isAllowed=y

5. Pokharel R, Silwal PR. Social health insurance in Nepal: A health system departure toward the universal health coverage. Int J Health Plann Manage. 2018 Apr 10;

6. Details of Districts where health insurance program has been implemented [Internet]. https://shs.gov.np/. 2024 [cited 2024 Mar 15]. Available from: https://hib.gov.np/en/detail/b-le-ll

7. Office of the Prime Minister and Council of Members Nepal. Health Insurance Regulation 2075. Nepal Government; 2019.

8. Government of Nepal, Ministry of Health. Nepal Health Sector Strategy Implementation Plan 2016-2021 [Internet]. Government of Nepal; 2017 [cited 2024 Mar 21]. Available from: https://www.aidsdatahub.org/resource/nepal-health-sector-strategy-implementation-plan-2016-2021

9. Government of Nepal, Social Health Security Development Committee. Annual Report of FY 2072/73 [Internet]. Available from: https://hib.gov.np/en/

10. Government of Nepal, Social Health Security Development Committee. Annual report of FY 2073/74 [Internet]. Available from: https://hib.gov.np/en/

11. Government of Nepal, Social Health Security Development Committee. Annual Report of FY 2074/75 [Internet]. Available from: https://hib.gov.np/en/

12. Government of Nepal, Social Health Security Development Committee. Annual Report of FY 2075/76 [Internet]. Available from: https://hib.gov.np/en/

13. Ranabhat CL, Subedi R, Karn S. Status and determinants of enrollment and dropout of health insurance in Nepal: an explorative study. Cost Eff Resour Alloc. 2020;18:40.

14. Acharya D, Devkota B, Wagle BP. Factors Associated to the Enrollment in Health Insurance: An Experience from Selected Districts of Nepal. Asian Social Science. 2019 Jan 30;15(2):p90.

15. Ghimire P, Sapkota VP, Poudyal AK. Factors Associated with Enrolment of Households in Nepal's National Health Insurance Program. International Journal of Health Policy and Management. 2019 Nov 1;8(11):636–45.

16. Shah S, Jha N, Khanal VK, Gurung GN, Sharma B, Shrestha M. Utilization of social health security scheme among the households of Illam district, Nepal. PLOS ONE. 2022 May 10;17(5):e0265993.

17. Castro FG, Kellison JG, Boyd SJ, Kopak A. A Methodology for Conducting Integrative Mixed Methods Research and Data Analyses. J Mix Methods Res. 2010 Sep 20;4(4):342–60.

18. Schoonenboom J, Johnson RB. How to Construct a Mixed Methods Research Design. Kolner Z Soz Sozpsychol. 2017;69(Suppl 2):107–31.

19. DHIS2. DHIS2 Overview [Internet]. [cited 2020 Oct 7]. Available from: https://www.dhis2.org/overview

20. Chun Tie Y, Birks M, Francis K. Grounded theory research: A design framework for novice researchers. SAGE Open Med [Internet]. 2019 Jan 2 [cited 2019 Apr 2];7. Available from: https://www.ncbi.nlm.nih.gov/pmc/articles/PMC6318722/

21. Home | Dedoose [Internet]. [cited 2021 Aug 30]. Available from: https://www.dedoose.com/

22. Ryan AM, Burgess JF, Dimick JB. Why We Should Not Be Indifferent to Specification Choices for Difference-in-Differences. Health Serv Res. 2015 Aug;50(4):1211–35.

23. Difference-in-Difference Estimation | Columbia University Mailman School of Public Health [Internet]. [cited 2018 Nov 10]. Available from: https://www.mailman.columbia.edu/research/population-health-methods/difference-difference-estimation

24. Imbens GM, Wooldridge JM. Recent Developments in the Econometrics of Program Evaluation [Internet]. National Bureau of Economic Research, Inc; 2008 Aug [cited 2018 Nov 10]. (NBER Working Papers). Report No.: 14251. Available from: https://ideas.repec.org/p/nbr/nberwo/14251.html

25. Stata 14 | Stata [Internet]. [cited 2021 Aug 30]. Available from: https://www.stata.com/stata14/

26. Normand CEM, Weber A, Carrin G. Social health insurance: a guidebook for planning. 2nd ed. Bad Homburg v.d.H: VAS, Verl. für Akademische Schriften; 2009. 158 p.

27. Obermann K, Jowett MR, Alcantara MOO, Banzon EP, Bodart C. Social health insurance in a developing country: the case of the Philippines. Soc Sci Med. 2006 Jun;62(12):3177–85.

28. Wang H, Zhang L, Yip W, Hsiao W. Adverse selection in a voluntary Rural Mutual Health Care health insurance scheme in China. Soc Sci Med. 2006 Sep;63(5):1236–45.

29. Obermann K, Jowett M, Kwon S. The role of national health insurance for achieving UHC in the Philippines: a mixed methods analysis. Glob Health Action. 2018;11(1):1483638.

30. Kwon S. Thirty years of national health insurance in South Korea: lessons for achieving universal health care coverage. Health Policy Plan. 2009 Jan;24(1):63–71.

31. The World Bank. GDP growth (annual %) | Data [Internet]. [cited 2018 Nov 22]. Available from: https://data.worldbank.org/indicator/NY.GDP.MKTP.KD.ZG?locations=NP

32. Paek SC, Meemon N, Wan TTH. Thailand's universal coverage scheme and its impact on health-seeking behavior. Springerplus. 2016 Nov 10;5(1):1952.

33. Carrin G, James C. Social Health Insurance: Key Factors Affecting the Transition Towards Universal Coverage. International Social Security Review. 2005 Jan 1;58(1):45–64.

34. Giedion U, Uribe MV. Colombia's Universal Health Insurance System. Health Affairs. 2009 May 1;28(3):853–63.

35. Gertler P, De Figueiredo R, Evans W, Hermalin B, Ghatak M, Blalock G, Herrin A. Who benefits from social health insurance? Evidence from the Philippines [Internet]. 2002 [cited 2018 Nov 14]. Available from: https://www.researchgate.net/publication/228427398_Who_benefits_from_social_health_insurance_Evidence_from_the_Philippines

36. Gertler P, Sturm R. Private health insurance and public expenditures in Jamaica. Journal of Econometrics. 1997 Mar 1;77(1):237–57.

37. Gertler PJ. On the road to social health insurance: the Asian experience. World Development. 1998 Apr 1;26(4):717–32.

Supporting Information

S1 Table. List of Intervention and comparator districts.

S2 File. Data collection instruments

S3 Figure. Graphical presentation of trends for client visits and referral outs from Primary Health Care Centers and Hospitals

Figure 1

Click here to access/download;Figure;Figure 1.tif

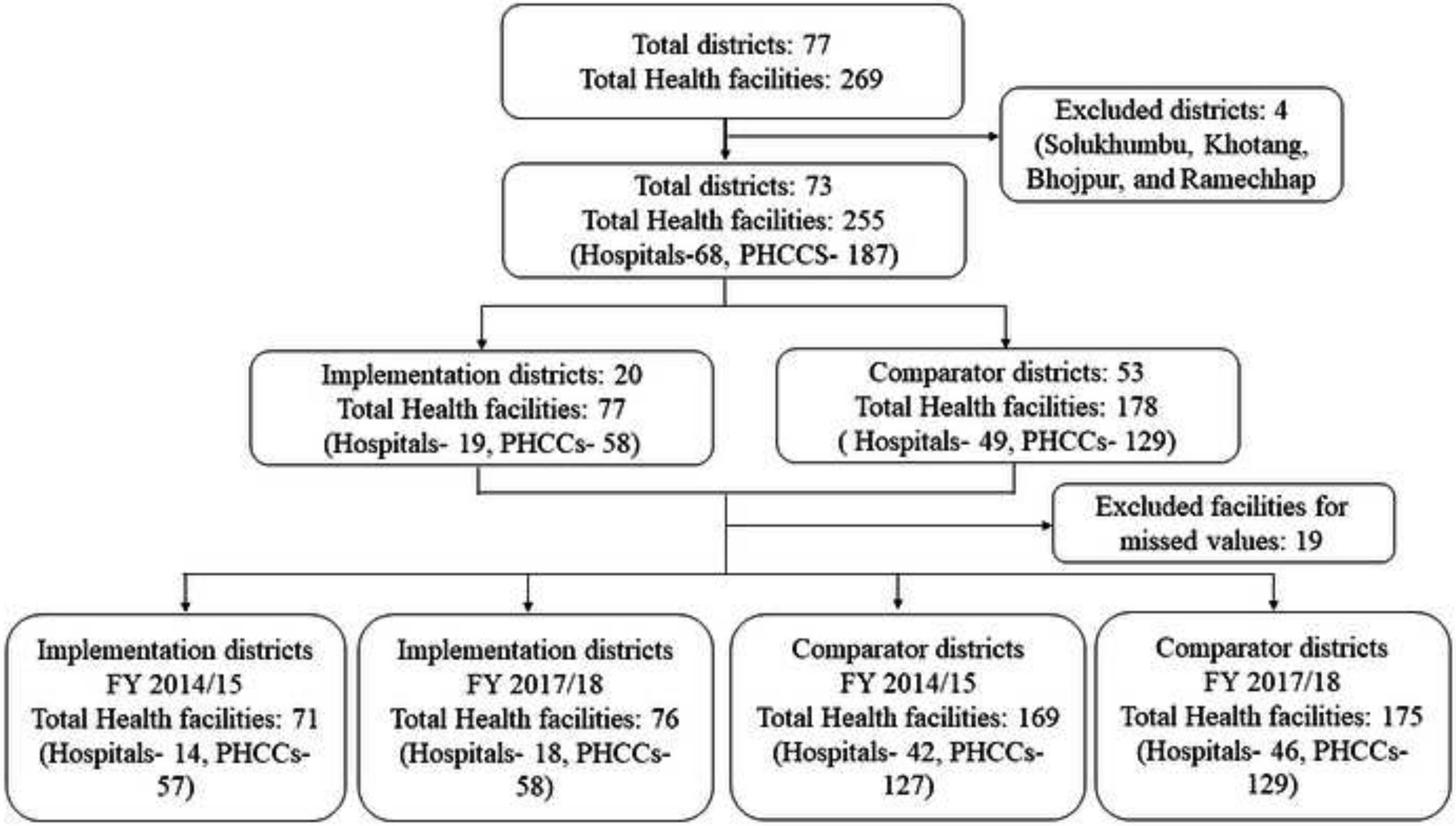

Figure 2

Click here to access/download;Figure;Figure 2.tif

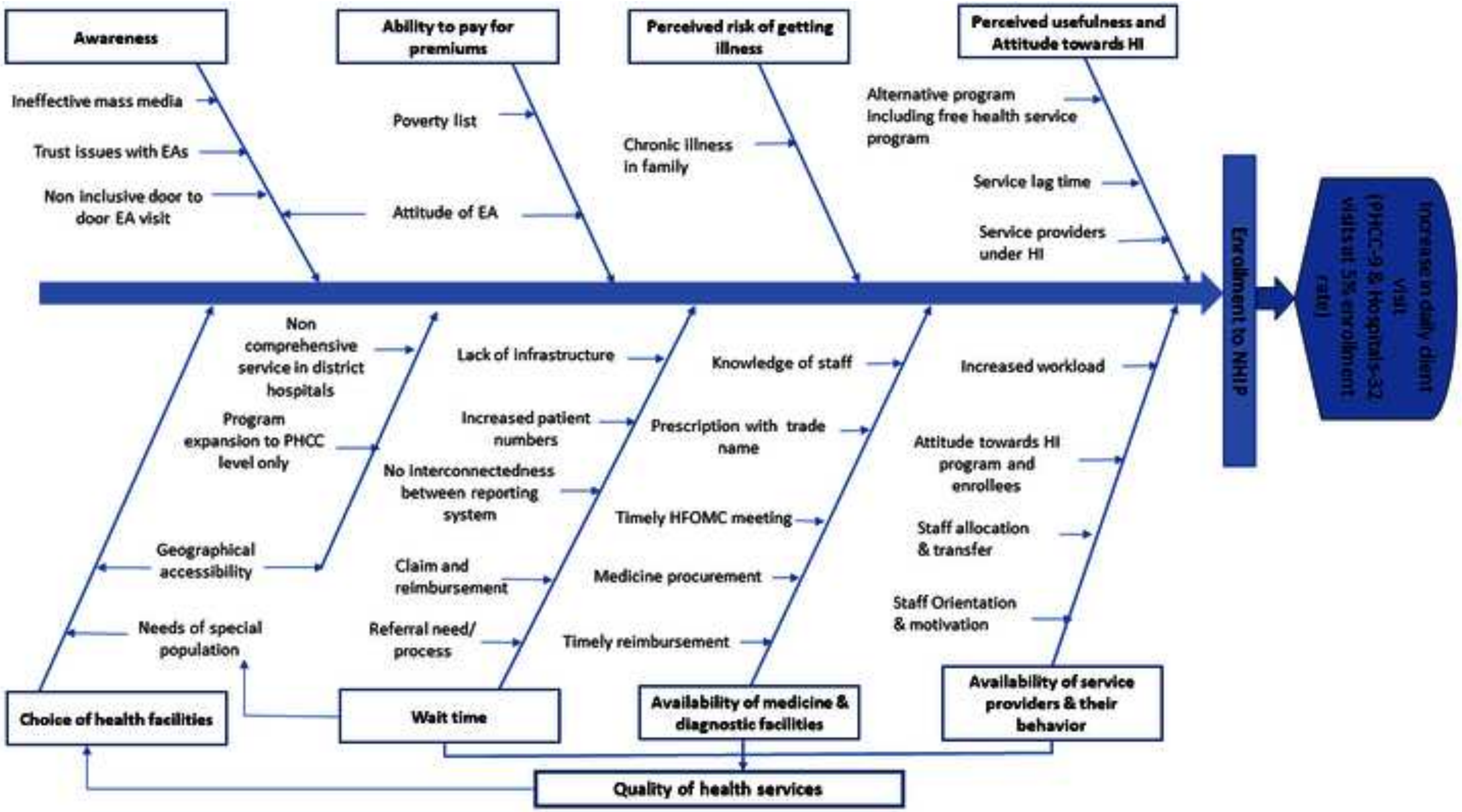

S1 Table

Click here to access/download
Supporting Information
S1. Table.docx

S2. File

Click here to access/download
Supporting Information
S2. File.docx

S3. Figure

Click here to access/download

Supporting Information - Compressed/ZIP File Archive

S3. Figure.zip
