# Supplementary material for: Experiences and effect of implementing social health insurance (SHI) program in Nepal-A mixed method study
Source: PLOS Glob Public Health. 2025 Apr 24;5(4):e0003492. doi: 10.1371/journal.pgph.0003492 (PMC12021158; doi:10.1371/journal.pgph.0003492)
Supplement: Attachment — Submitted filename: PGPH-D-24-00962-NEPAL health insurance.docx [file pgph.0003492.s005.docx]

PLOS GLOBAL PUBLIC HEALTH

REVIEWERS COMMENTS

PGPH-D-24-00962: Experiences and effect of implementing Social Health Insurance (SHI) program in Nepal-A mixed method study

Dear Editor,

I submit to you my comments on the above manuscript submitted to PGPH for review

**General comments**

Overall, the intentions of the manuscript and the issues discussed are relevant. However, the authors need to place the results in perspective to enhance its understanding.

**Specific comments**

I provide some specific comments here, which will include comments and questions.

1. The presentation of the results section of the abstract is very general. Per the research objectives, the authors should present the results accordingly i.e. providers, client and then the effect.
2. Again in the abstract, there was no results from the DID analysis for the effect of enrolment on utilization.
3. Abstract: the conclusion seems not to emanate from the findings of the research. There were specific client and provider results to which the study can conclude on as well as make recommendations. The current conclusion is focusing on collaboration between the Health Insurance Board and the Ministry. These are important but they do not the direct results from the study or the objectives to which the study was set.
4. Study objectives: Lines 133-138. I suggest that the objectives are not numbered. They could be considered as a small paragraph at the end of the introduction
5. ‘FY’…please write in full for the first time it appears in the manuscript
6. Methods: What informed the use of the exploratory sequential mixed methods design? In my simple understanding of the sequence it implies qual-quant-qual sequence. However, per the data collection, analysis and results presented, it seems like the first two objectives followed the qualitative approach while the last objective followed the quantitative approach.
7. It does not look like the qualitative informed the quantitative approach, and neither did the quantitative approach added information to the qualitative. Per the way the results have been presented. That linkage is missing
8. From lines 244 in the results section, the themes were just presented. The reader is not lead to know what exactly is going to be discussed. Do they relate to the first objective? Similarly as you read along, then the findings that relate to the providers experience also follow but the reader is not guided. For instance I will expect that before for example line 244, you introduce briefly the major themes that emanated from the clients experience and then the details will follow. This should be the same for the providers
9. I also did not see any connection between the qualitative findings and the quantitative findings. If there are no connections or the studies were conducted independently, then this should be stated clearly and a revision of the type of mixed methods study design
10. Lines 270-272: the quote relates to human resource skills than availability of medicines and diagnostic facilities per the theme.
11. Major findings and key issues in the study did not come out clearly. I find some of the issues addressed in the discussion not coming from the results-adverse selection, financial risk protection for example are two such issue that was not clearly indicated in the results.

Thank you
