# Supplementary material for: Experiences and effect of implementing social health insurance (SHI) program in Nepal-A mixed method study
Source: PLOS Glob Public Health. 2025 Apr 24;5(4):e0003492. doi: 10.1371/journal.pgph.0003492 (PMC12021158; doi:10.1371/journal.pgph.0003492)
Supplement: Attachment — Submitted filename: Response to Reviewers.docx [file pgph.0003492.s006.docx]

Response to reviewers’ comments

| Reviewers’ comments | Authors’ responses | Change in manuscript |
| --- | --- | --- |
| **Reviewer 1** |  |  |
| **Introduction** |  |  |
| After reading this, I’m still left wondering about the history of policies, and what is included, how is it delivered, and why there is a need for enrolment as opposed to automatic universal enrolment. More details and clarity of the process will be helpful. | *Health Insurance Act 2074 has envisioned automatic universal enrolment and has made additional provisions on current voluntary enrolment. However, these additional provisions have yet to be implemented.* | *The change has been added under “population coverage” within NHIP design features* |
| Why is public financing alone not feasible in Nepal? Please add a line or two on this to hook onto global health discussions on this topic. | *The context has been added to the introduction section* | Page 7, line 126-128 |
| The introduction section will benefit from more discussions of published international studies on the pros and cons of social insurance systems, and why it is the policy option chosen in national circumstances, and what are known challenges and enablers in other low and middle income countries. | *The global context has been added to the introduction section* | Page 6-7, line 100-112 |
| **Methods** |  |  |
| To clarify- was on overall grounded theory method used for the study including identification of participants? Or just in analysis? Please clearly describe this, and why/how did the grounded theory analysis be considered suitable, and what were the novel theories or themes identified from this process? How did the analysis method differ from thematic analysis etc? | *The grounded theory was used for both data collection and analysis. At the time of data collection, only a few studies had explored the challenges of the demand and supply side in implementing NHIP. So, we considered the grounded theory approach. The information obtained during data collection was used to reformulate the recruitment and data collection strategies. For instance, the location where we held the focus groups was deliberately chosen to capture the thoughts of enrolees and non-enrolees living closer and distant to health facilities.*  *The context has been added to the paper.* | Page 12, Line 178- 187 |
| **Results and Discussions** |  |  |
| On the demand side, how did demographic and intersectionality affect enrolment, use of services, and drop out? Why? You’ve provided the categories represented in research, but not so much details of how these categories influenced demand-related perceptions and decisions. | *We have added additional details in the discussion section.* | Page 24-25, line 431- 442 |
| How were themes distinctly related to the design and implementation of social insurance program as opposed to broader health system factors- for instance- you mention quality as one of the biggest consideration, but how is that tied to social insurance design and implementation, as opposed to broader health system factors? Related procurement policies, and service quality. Please describe and discuss. | *Themes such as the quality of health services have always been important health system factors. However, they have been worsened by the increased patient load and administrative burden following the implementation of the NHIP. The tendering process for medicine procurement is the requirement of NHIP.*  *The clarifying language has been added to the manuscript.* | Page 17, line 274-276 |
| Perhaps it is outside the scope of the results but worthy of a discussion or identification of future research= how are demand related factors influenced by age, gender, education, employment status and income levels, etc? Who precisely are benefitting, who are not and dropping out? For instance- gender and social insurance or employment related health entitlements is an area that can influence demand or rather even when there is demand, the ability of continuously access timely and necessary health care- see these publications for context: | *Has been added to the discussion section* | Page 24-25, line 431-442 |
| **Reviewer 2** |  |  |
| **Abstract** |  |  |
| The presentation of the results section of the abstract is very general. Per the research objectives, the authors should present the results accordingly i.e. providers, client and then the effect. | *The abstract has been rewritten* | Page 4-5 |
| Again in the abstract, there was no results from the DID analysis for the effect of enrolment on utilization. | *The findings from DID have been added* | Page 5 |
| the conclusion seems not to emanate from the findings of the research. There were specific client and provider results to which the study can conclude on as well as make recommendations. The current conclusion is focusing on collaboration between the Health Insurance Board and the Ministry. These are important but they do not the direct results from the study or the objectives to which the study was set. | *The conclusion is rewritten for more clarity. Changes have been made in the discussion section.* | Page 5 |
| **Study Objective** |  |  |
| Lines 133-138. I suggest that the objectives are not numbered. They could be considered as a small paragraph at the end of the introduction. | *The numbers are removed,* | Page 8, Line 143-147 |
| ‘FY’…please write in full for the first time it appears in the manuscript | *Added* | Page 7, Line 133 |
| **Methods** |  |  |
| What informed the use of the exploratory sequential mixed methods design? In my simple understanding of the sequence it implies qual-quant-qual sequence. However, per the data collection, analysis and results presented, it seems like the first two objectives followed the qualitative approach while the last objective followed the quantitative approach. | *The exploratory* *sequential* mixed-method design *involves qualitative data collection, qualitative data analysis, qualitative findings inform quantitative data collection, quantitative data analysis, and merging and join display. Our study started with qualitative data collection. Since qualitative findings suggested the increase in patient flow and referrals in the health facilities, those were ascertained through quantitative findings using DID method. The findings were jointly displayed using the fishbone diagram.* | Page 10-11, Line 151-156 |
| It does not look like the qualitative informed the quantitative approach, and neither did the quantitative approach added information to the qualitative. Per the way the results have been presented. That linkage is missing | *The quantitative approach provided quantifiable information to our qualitative findings suggesting increase in referrals and patient flow.* | Page 23-24  Line 413- 430 |
| From lines 244 in the results section, the themes were just presented. The reader is not lead to know what exactly is going to be discussed. Do they relate to the first objective? Similarly as you read along, then the findings that relate to the providers experience also follow but the reader is not guided. For instance I will expect that before for example line 244, you introduce briefly the major themes that emanated from the clients experience and then the details will follow. This should be the same for the providers | *A paragraph has been added with the themes identified from demand and supply side.* | Page 16-17, line 253-263  Page 23-24  Line 413- 430 |
| I also did not see any connection between the qualitative findings and the quantitative findings. If there are no connections or the studies were conducted independently, then this should be stated clearly and a revision of the type of mixed methods study design | *The qualitative findings suggested the increase in patient load, referrals, and administrative burden leading to various challenges. The variables for quantitative analysis were identified based on the qualitative findings. The quantitative portion verifies and quantifies the qualitative findings.*  *The language has been clarified in the manuscript.* | Page 16-17, line 253-268  Page 23-24  Line 413- 430 |
| Lines 270-272: the quote relates to human resource skills than availability of medicines and diagnostic facilities per the theme. | *The quote has been changed.* | Page 17-18, Line 278-282 |
| Major findings and key issues in the study did not come out clearly. I find some of the issues addressed in the discussion not coming from the results-adverse selection, financial risk protection for example are two such issue that was not clearly indicated in the results | *Adverse selection and financial risk protection are the implications that our findings suggested. Our results has shown that households are partially participating, often enrolling high-risk or chronically ill members. We also see high enrolment among those families with chronically ill members. These assessments implied the adverse selection.*  *Likewise, with benefit ceiling of NPR 100,000 and people opting for private hospitals, in addition to private facilities charging gap amount from insurance enrollees does implicate against financial risk protection.* |  |
| Results: |  |  |
| Page 16: For ease of reading, suggest to sign-post the difference between supply and demand side themes. | *A paragraph has been added with demand side and supply side themes. As these are intricately connected with some being mutually dependant and others being consequence of each other, we have captured this complex relationship in a fish-bone diagram (Figure 2).* | Page 16-17, Line 253- 268 |
| Page 17: How would you tie this back to social insurance design and implementation? | *The patient load and the administrative burden were reported to be increased after NHIP implementation. NHIP requires health facilities to have their own pharmacy, medicine procurement process and data reporting mechanism. However, there are no additional sanctioned position to fulfil these responsibilities. Changes in the manuscript has been made to reflect this.* | Page 19, Line 310-317 |
| Page 24: Ability to Pay Premiums: this theme is related the design of social insurance- will be useful to have more description and elaboration. | *Means testing is not under NHIP. Nepal government had created a poverty list based on poor identification survey. But many participants claim that the list is not accurate. Description of the survey has been added.* | Page 22, line 377-380 |
